# Predicting Arsenic (As) Exposure on Human Health for Better Management of Drinking Water Sources

**DOI:** 10.3390/ijerph18157997

**Published:** 2021-07-28

**Authors:** Minhaz Farid Ahmed, Chen Kim Lim, Mazlin Bin Mokhtar, Rd. Puteri Khairani Khirotdin

**Affiliations:** Institute for Environment and Development (LESTARI), Universiti Kebangsaan Malaysia (UKM), Bangi 43600, Malaysia; kim@ukm.edu.my (C.K.L.); rdputeri@ukm.edu.my (R.P.K.K.)

**Keywords:** GBT model, predictive analysis, arsenic, Langat River Basin, Malaysia, health risk, drinking water

## Abstract

Chemical pollution in the transboundary Langat River in Malaysia is common both from point and non-point sources. Therefore, the water treatment plants (WTPS) at the Langat River Basin have experienced frequent shutdown incidents. However, the Langat River is one of the main sources of drinking water to almost one-third of the population in Selangor state. Meanwhile, several studies have reported a high concentration of Arsenic (As) in the Langat River that is toxic if ingested via drinking water. However, this is a pioneer study that predicts the As concentration in the Langat River based on time-series data from 2005–2014 to estimate the health risk associated with As ingestion via drinking water at the Langat River Basin. Several time-series prediction models were tested and Gradient Boosted Tree (GBT) gained the best result. This GBT model also fits better to predict the As concentration until December 2024. The mean concentration of As in the Langat River for both 2014 and 2024, as well as the carcinogenic and non-carcinogenic health risks of As ingestion via drinking water, were within the drinking water quality standards proposed by the World Health Organization and Ministry of Health Malaysia. However, the ingestion of trace amounts of As over a long period might be detrimental to human health because of its non-biodegradable characteristics. Therefore, it is important to manage the drinking water sources to minimise As exposure risks to human health.

## 1. Introduction

The presence of toxic arsenic (As) in the environment, especially in the aquatic environment, is very detrimental to all living organisms due to its persistent and non-biodegradable characteristics [1,2,3,4,5]. Meanwhile, globally, many studies have reported As exposure risks to human health via environmental media, especially As ingestion via drinking water and dietary consumption [4,5,6,7,8,9,10,11,12,13]. Arsenic in the environment is mainly from natural sources such as weathering of mineral rocks that contributed to the hydrochemistry of the river [14,15,16]. Apart from natural sources, industrialization, urbanization, and other anthropogenic activities have also contributed to the As concentration in the environment and enhanced the As exposure risks to human health [17]. In the Langat River Basin in Malaysia, several studies have also reported a high concentration of As in the river both from natural and man-made sources. For instance, the mean concentrations of As were reported in the Langat River by Ahmed et al. [6], 1.65 ± 0.93 μg/L (range 0.33–3.04 μg/L); Aries et al. [14], 11.18 ± 8.29 μg/L (range 1.79–21.48 μg/L); Sarmani [18], 201.11 μg/L (range 90–330 μg/L), and Yusuf [19], 27.50 μg/L. Therefore, examining the water quality in terms of As contamination in the Langat River is very important because it provides drinking water to almost one-third of the population in Selangor state. It is assumed that the health risk of As ingestion via drinking water reduces along with the reduction of As concentration in the drinking water sources. Thus, a prediction study based on the time-series data of As concentration will also contribute to managing the pollution reduction in the Langat River as well as reducing the As exposure risks to human health.

The river quality in terms of the Water Quality Index (WQI) showed an increase in 2019 around Malaysia. Out of the 672 rivers monitored, 61% showed clean water quality in 2019. The percentage of slightly polluted rivers and polluted rivers were 30% and 9%, respectively [20]. However, there are many branches of the Langat River in Selangor state and the maximum of these branches were slightly polluted, i.e., class III that requires extensive treatment before drinking. The average WQI of the Langat River also ranged from ‘slightly polluted’ to ‘clean’ during 2003–2019 [20,21,22,23]. Although the upstream water quality of the Langat River was clean, the physico-chemical parameters including several metals in so many cases had crossed the national water quality index at mid to downstream. Additionally, the upstream of Langat River also recorded a high concentration of several metals such as aluminium, arsenic, cadmium, chromium, lead, etc., mainly from natural sources [6,24,25,26,27]. Therefore, several water treatment plants (WTPs) such as Sg. Semenyih, Sg. Langat, Cheras Mile 11, Bukit Tempoi, Salak Tinggi WTPs from 2009 to 2020 have experienced frequent shutdown incidents due to the chemical pollutions in the Langat River [25,28]. The sources of this pollution were both point and non-point sources such as the effluent from industrial zones and animal husbandries, leachate from landfills, run-off from the palm oil plantation and agriculture activities, frequent flash floods due to climate change, and similar [25,29].

The land-use and land cover changes due to rapid urbanization and industrialization in South East Asia have also altered the underground geochemistry and enhanced the mobilization of As [30]. The dominant soil types in the Langat River Basin such as oxisols and ultisols are also the natural sources of As in the Langat River, apart from the mining and navigation activities, as well as the use of arsenal herbicides in the agricultural activates [6,14,18].

Therefore, the leadership roles of local governments to enforce the laws effectively via the functional multi-stakeholders’ platform is very important to reduce the pollution in the river both from point and non-point sources [24,31,32]. Ahmed et al. [26] have also emphasized the capacity-building of relevant stakeholders via awareness-raising, advocacy, and appropriate training using the multi-stakeholder platforms for decision making in the river basin management, including reducing pollution in line with the integrated water resources management (IWRM). However, the inadequate collaboration and cooperation among all the relevant stakeholders have been a challenge to manage the transboundary Langat River, especially for the pollution management [26,31,33,34]. Therefore, scientific data and information such as the prediction model of As pollution in the Langat River can contribute significantly to the decision-making processes of the local government for pollution reduction in the Langat River because it has the mandate based on the ‘Local Government Act 1976’. Thus, this study used the time-series data of As concentration in the Langat River (2005–2014) and predicted As concentration until December 2024 along with estimating the As exposure risks to human health via ingestion to suggest effective leadership roles of the relevant stakeholders for better drinking water management.

## 2. Study Area

Langat River Basin is one of the important river basins in the Selangor state of Malaysia which covers approximately an area of 2409 km^2^ [21] within the latitudes 2°40′152″ N to 3°16′15″ N and longitudes 101°19′20″ E to 102°1′10″ E [35,36]. In the upstream of Langat River Basin is the Titiwangsa granite mountain range where the Langat River originates at the Gunang Nuang and drains about 190 km via the Selangor and Negeri Sembilan states, as well as the Federal Territories of Putrajaya and Kuala Lumpur until it falls in the Strait of Malacca (Table 1).

In the Langat Basin, there are two dams, Semenyih and Langat, which are potential reservoirs of drinking water. Apart from these dams, there are many ex-mining ponds all over the basin, especially at the Kuala Langat near the Paya Indah Wetlands. The topography of the basin is defined as both mountainous and flat, and the average elevation ranges from 1440–400 m. The elevation of the central basin on average is below 200 m followed by less than 100 m at the lower basin. The rock beneath the Langat River Basin is igneous rock that is mainly granite. Therefore, the geology of the basin is defined as Hawthornden Schist and Kenny Hill Formation (sandstone and phyllite). Tanah Curam (Steepland), Rengan-Jerangan (urban land), and Tanah Gambut (peat soils) are the main soil types in the hilly upstream, flat midstream, and downstream, respectively. The average annual rainfall at the Langat River Basin is about 2483.62 ± 269.70 mm ranging from 2043.68 mm to 2832.40 mm during 2005–2016 [24,31,37].

## 3. Methods

### 3.1. Data Collection

**Figure 1:**
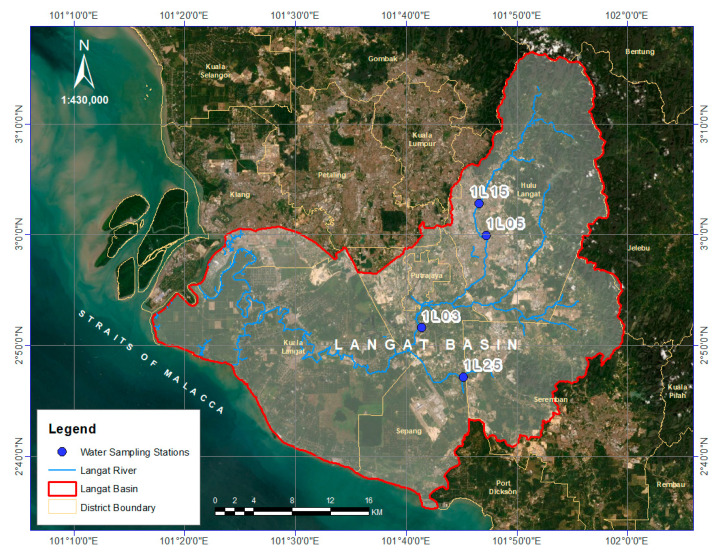
Water sampling stations at the Langat River Basin, Malaysia.

Monthly water quality data of As (μg/L) was collected from the four water quality monitoring stations of the Department of Environment Malaysia (DOE) in the Langat River (Figure 1) from January 2005 to December 2014. Accordingly, the monthly data of physico-chemical parameters such as dissolved oxygen (DO, mg/L), pH, temperature (Temp, °C), salinity (SAL, ppt), and dissolved solids (DS, mg/L) were also obtained (2005–2014) from the same stations of the Department of Environment Malaysia. The monthly missing values during 2005–2014 were replaced with the yearly mean value of As and physico-chemical parameters data. The mean value of obtained As and physico-chemical parameters were found significant at a 95% confidence interval via the one-sample *t*-test (Table 2).

SPSS software (Version 21.0, IBM Corp., Armonk, NY, USA) was applied to perform the descriptive statistics of As concentrations and physico-chemical parameters. The descriptive statistics include the calculation of the minimum (min.), maximum (max.), mean, and standard deviation (Std. Dev.) of the concentration of the water quality parameters in the Langat River. The standard deviation was calculated to observe the precision of each water quality parameter. Accordingly, Microsoft Excel 2016 (Microsoft Office Professional Plus 2016, Microsoft Corporation, Redmond, Washington, USA) was used to produce the trend graphs of As and the physico-chemical parameters during 2005–2014 of the Langat River. Pearson’s statistical correlation analysis was applied to estimate the correlations among the As concentrations and physico-chemical water quality parameters.

### 3.2. Prediction Model for Arsenic Concentration

**Figure 2:**
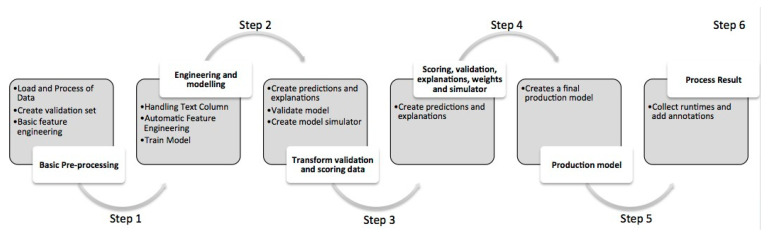
Six steps of the Gradient Boosted Trees (GBT) model’s processes.

During the process of modelling to predict, RapidMiner Studio (Version 9.8, RapidMiner, Inc., Boston, MA USA) was used. There are six models, namely Generalized Linear Model, Deep Learning, Decision Tree, Random Forest, Gradient Boosted Trees (GBT), and Support Vector Machine (SVM) that give the best top six results. Out of the six models, GBT has the best gain and best performance. The six steps of GBT processes (i.e., details in Supplementary Material: Tables S1–S3, Figures S1 and S2) are generalized as follows (Figure 2) solely to run this set of data for QA (i.e., quality assurance) and QC (i.e., quality control) purposes.

Friedman [38] proposed the Gradient Boosted Trees (GBT) model in 2001. GBT produces competitive, highly robust, interpretable procedures for both regression and classification, especially appropriate for mining less than cleaning data [38,39]. The gradient boosted tree (GBT) algorithm performed best on both the validation set and the challenge test set. For instance, the GBT algorithm, which outperformed other models on both the validation set and the challenge test set, is also used for 2017’s Soccer Prediction Challenge [39], multi-solar power forecasting [40], and clinical mastitis based on milking data set [41].

### 3.3. Human Health Risk Assessment 

The following equations of the United States Environmental Protection Agency [42,43] have been used to assess the human health risk of As ingestion via drinking water from the Langat River. These equations have also been used globally by researchers to assess As exposures’ risks to human health [6,44,45,46,47,48,49].

(1) $$CDI\;\left(\frac{\mathrm{mg}}{\mathrm{kg}}/\mathrm{Day}\right)=\frac{Cdw\;\left(\frac{mg}{L}\right)\times IR\;\left(\frac{L}{Day}\right)\times EF\;\left(\frac{Day}{Year}\right)\times ED\;\left(Years\right)}{BW\;\left(kg\right)\times AT\;\left(Days\right)}$$

(2) $$HQ=\frac{CDI\;\left(\frac{\mathrm{mg}}{\mathrm{kg}}/\mathrm{Day}\right)}{RfD\;\left(\frac{\mathrm{mg}}{\mathrm{kg}}/\mathrm{Day}\right)}$$

(3) $$LCR=CDI\;\left(\frac{mg}{kg}/Day\right)\times SF{\left(\frac{mg}{kg}/\mathrm{Day}\right)}^{-1}$$

here, *CDI* = chronic daily intake (mg/kg-Day); *Cdw* = mean concentration of metal in water (mg/L); *IR* = water ingestion rate 1.996 L/Day at Langat River Basin [6]; *EF* = exposure frequency 365 Days/Year [6,43]; *ED* = exposure duration 74 Years [6,50]; *BW* = body weight 63.193 kg [6]; *AT* = average time 27,010 Days [43,50]; *HQ* = hazard quotient; *RfD* = chronic reference dose of arsenic 3.0 × 10^−4^ [51]; *LCR* = lifetime cancer risk; *SF* = slope factor 1.5 of arsenic [51].

## 4. Results and Discussion

### 4.1. Arsenic Concentration in Langat River

The mean As concentration, 3.73 ± 1.97 μg/L (Table 3), in the Langat River was within the river and drinking water quality standards proposed by the Ministry of Health (MOH) 10 μg/L, the World Health Organization (WHO) 10 μg/L, and the United States Environmental Protection Agency (USEPA) 150 μg/L. However, the mean range of As, 0.98–12.87 μg/L, showed that the maximum mean As concentration had exceeded the maximum limit of MOH and WHO, respectively, 10 μg/L. Among all the water-sampling stations, the 1L15 station at the upstream showed the highest maximum concentration of As, 21.94 μg/L followed by As, 15.04 μg/L at the 1L05 station. On the other hand, the mean values of physico-chemical parameters, DO 6.24 ± 0.86 mg/L, pH 12.63 ± 0.67, temp 20.96 ± 1.05 °C, SAL 9.43 ± 3.15 ppt, and DS 53.14 ± 27.86 mg/L, were within the class I category proposed by the Dept. of Environment [52]. Among the stations, the 1L25 station at the downstream recorded a higher value of pH 29.46 ± 1.74 and SAL 37.46 ± 12.33 ppt than the other stations (Table 3).

**Figure 3:**
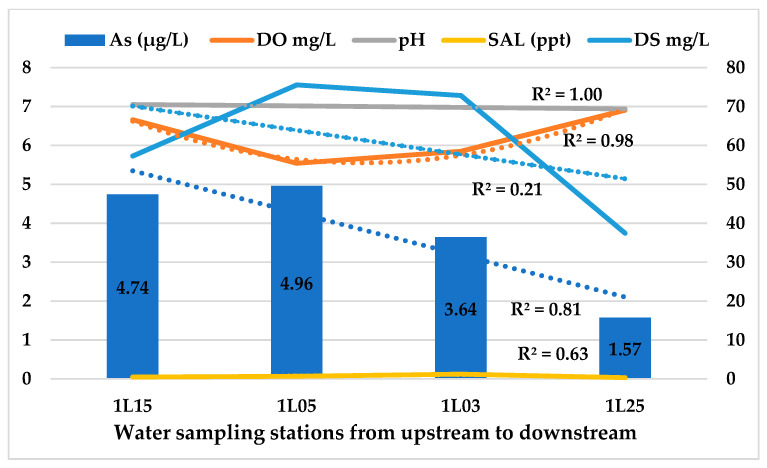
As (μg/L) concentration from upstream to downstream in the Langat River.

The decreasing trend (R^2^ = 0.81; Figure 3) of As concentration from upstream to downstream in the Langat River might be due to the higher level of salinity 37.46 ppt (Table 3) towards downstream (R^2^ = 0.63). The increasing trend of DO (R^2^ = 0.98) might have also enhanced the As mobility and its precipitation on sediments along with other salts. Moreover, a moderate negative correlation between As concentration and DO (r = −0.524, *p* = 4.2 × 10^−10^) was found in the Langat River at a 99% confidence level (Table 4). Other studies have also reported a lower level of As concentration at the downstream of the Langat River than the upstream [6,57,58], which might be due to the proximity of water sampling stations towards the sea, i.e., Straits of Malacca where the Langat River meets the Indian Ocean.

**Figure 4:**
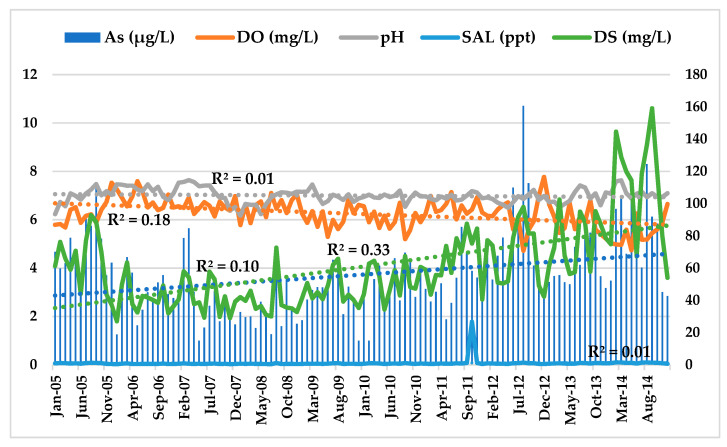
As (μg/L) concentration from 2005 to 2014 in the Langat River.

The higher level of salts in downstream than in upstream increases the mobility of As and its precipitations on the sediments. However, the higher level of As in the upstream 1L15 4.74 ± 2.87 μg/L (Figure 4) might be because of the natural weathering of arsenopyrite minerals in the Titiwangsa mountain range [6,58]. The man-made activities along with the non-point sources of pollution might have also increased As concentration in the stations of midstream 1L05 As 4.96 ± 2.57 μg/L. Effluent from the sewage treatment plants and industries, leachate from the landfills and animal farms, and such are the point sources of pollution in the Langat River. However, runoff of arsenal herbicides from the agricultural and palm oil plantation areas, especially via the frequent flash floods, are the main non-point sources of As pollution in the Langat River [6,29,59].

Accordingly, the temporal distribution of As concentration in the Langat River from 2005–2014 (Figure 4) showed a little bit of an increasing trend (R^2^ = 0.10) along with the increasing trend of DS (R^2^ = 0.33). These indicate that higher runoff of nutrients in the Langat River might have been due to the huge forest and land clearance activities for rapid urbanization and industrialization at the basin [29,31]. Moreover, the Pearson correlation analysis also showed that As concentration in the Langat River has a very strong positive correlation with DS (r = 0.704, *p* = 1 × 10^−13^) and temp. (r = 0.229, *p* = 0.006) at a 99% confidence level, respectively (Table 4).

### 4.2. Prediction Model on Arsenic Concentration in Langat River

**Figure 5:**
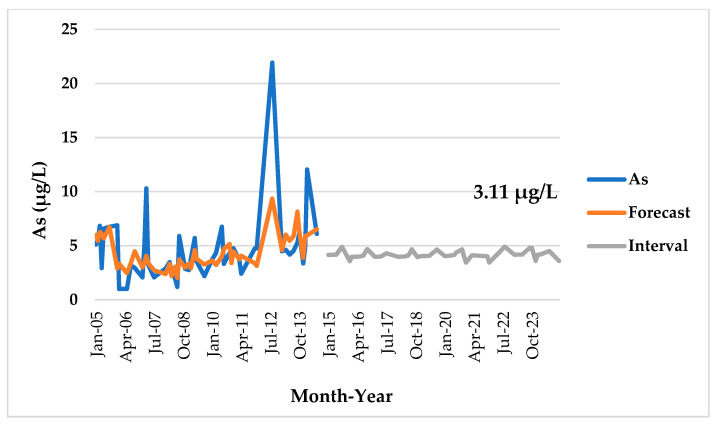
Forecast of As (μg/L) concentration in the Langat River based on the GBT model from January 2005 to December 2024.

The GBT model predicted an As concentration of 3.11 μg/L for December 2024 (Figure 5) based on the monthly time-series data of As (μg/L) concentration from 2005–2014 in the Langat River. However, the mean concentration of As, 3.11 μg/L, for December 2024 is lower than the concentration of As 3.73 ± 1.97 μg/L at December 2014. This indicates the better river basin management practices by the entire relevant stakeholders, especially to reduce the river pollution. It is also noted that the Academy of Sciences Malaysia (ASM), which is a strategic partner of the government of Malaysia, has produced the ‘Transforming the Water Sector: National Integrated Water Resources Management Plan, Strategies and Road Map’ in 2016. This IWRM plan is currently at the implementation level and could bring good results for the river basin management in Malaysia [59].

**Figure 6:**
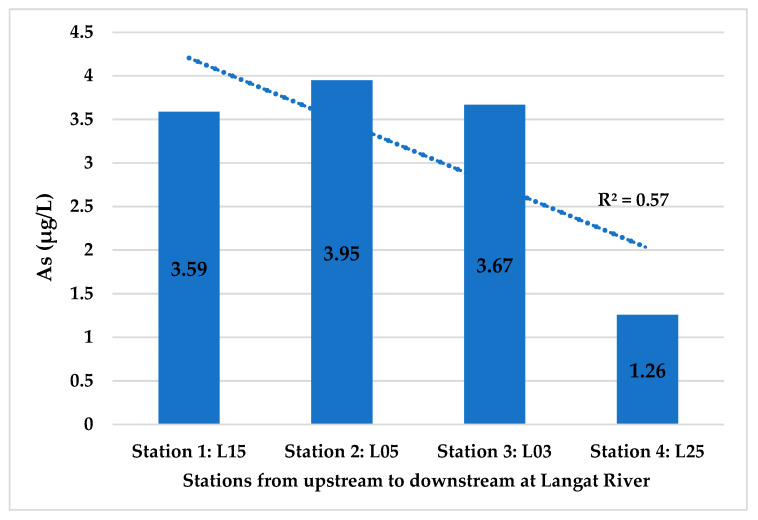
Prediction of As (μg/L) concentration at water stations for December 2024.

Ahmed et al. [6] also forecasted an As concentration of 3.45 μg/L (i.e., for January 2020) in the Langat River based on the monthly data from January 2005 to August 2015 using the auto-regression moving average analysis. However, the forecast of the As concentration of 3.45 μg/L at January 2020 by Ahmed et al. [6] was also lower than the determined mean As concentration of 3.55 ± 1.75 μg/L in Langat River in that study from January 2005 to August 2015. Similarly, the forecast of As concentration, 3.11 μg/L (i.e., for December 2024), in this study was also lower than the mean As concentration of 3.73 ± 1.97 μg/L from January 2005 to December 2014. Moreover, As concentration at the four sampling stations in this study from upstream to downstream of the Langat River also showed a moderate decreasing trend (R^2^ = 0.57) based on the data 2005–2014, which might be due to the dissolution of As concentration towards downstream because of the high level of salinity (Figure 6).

### 4.3. Health Risk of Arsenic Ingestion via Drinking Water

**Figure 7:**
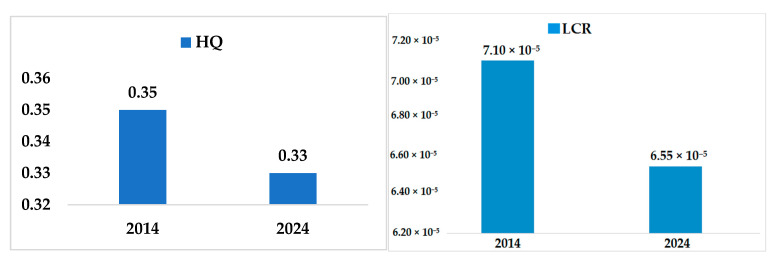
Predicted HQ and LCR of As ingestion via drinking water from Langat River in 2014 and for 2024, respectively.

This study calculated that the long-term As ingestion via drinking water from the Langat River has no potential carcinogenic and non-carcinogenic health risk. The carcinogenic lifetime cancer risk (LCR) has been calculated at 7.10 × 10^−5^ in 2014 and 6.55 × 10^−5^ for 2024 (Figure 7). LCR 10^−5^ indicates the risk of one additional occurrence of cancer in one hundred thousand people [42]. Similarly, the non-carcinogenic hazard quotient (HQ) has been calculated at 0.35 in 2014 and 0.33 for 2024, which are less than 1. Any value of HQ less than 1 is safe from being a non-carcinogenic health risk. The previous study also reported no potential carcinogenic (i.e., LCR 9.7 × 10^−6^) and non-carcinogenic (i.e., HQ 4.8 × 10^−2^) health risks of As ingestion via drinking water in the Langat River Basin in 2015. However, the association has been indicated between long-term As ingestion via drinking water, and kidney failure and liver cancer in the Langat River Basin [6]. Therefore, further study is required to link the causal relationship between As ingestion via drinking water and human health risk at the Langat River Basin to reduce the health burden.

## 5. Conclusions

The prediction of the As concentration of 3.11 μg/L for 2024 following the GBT model was within the drinking water quality standards of the Ministry of Health (MOH), the World Health Organization (WHO), and the United States Environmental Protection Agency (USEPA), respectively. The prediction of As concentration for 2024 was based on the monthly time-series data from 2005 to 2014. Moreover, the determined As concentration of 3.73 ± 1.97 μg/L at 2014 in the Langat River was also within the drinking water quality standards of MOH, WHO, and USEPA. Therefore, the predicted carcinogenic and non-carcinogenic health risks of As ingestion via drinking water at the Langat River Basin showed no potential health risks both for 2014 (HQ 0.35; LCR 7.10 × 10^−5^) and 2024 (HQ 0.33; LCR 6.55 × 10^−5^). Thus, the health risk of As ingestion via drinking water is reduced along with the reduction of As concentration for 2024 compared with 2014. This indicates the better management of the Langat River, including the pollution reduction via the effective implementation of IWRM policies along with the empowerment of the local government for its leadership roles.

Although the current implementation of the policy such as ‘Transforming the Water Sector: National Integrated Water Resources Management Plan, Strategies and Road Map’ is contributing to better IWRM, however, the effective implementation of policies should prioritize addressing specific units in IWRM such as the river basin management authority. Because several local authorities are functioning within the Langat River Basin, the river basin management authority could coordinate better with all the local authorities for total water management including the issues of transboundary river pollution. Moreover, a further causal study is required to link the causal relationship between As ingestion via drinking water and human health risk at the Langat River Basin to suggest reducing As exposure risks to human health. Hence, extensive studies on water treatment technologies at both the water treatment plant and household water filtration levels are required to ensure a safe drinking water supply at the Langat River Basin.

## Figures and Tables

**Table 1:** Administrative areas of Langat River Basin, Malaysia.

State	District	Area (km^2^)	Local Authority
Selangor	Klang	71.87	Majlis Perbandaran Klang (MPK)
	Kuala Langat	706.93	Majlis Daerah Kuala Langat (MDKL)
	Sepang	333.25	Majlis Perbandaran Sepang (MPS)
	Hulu Langat	809.34	Majlis Perbandaran Kajang (MPKj)
Negeri Sembilan	Seremban	445.12	Majlis Perbandaran Nilai (MPN)
W.P. Putrajaya	-	39.21	Perbadanan Putrajaya (PPj)
W.P. Kuala Lumpur	-	3.67	Dewan Bandaraya Kuala Lumpur (DBKL)
	Total Area	2409.39	

Source: [21].

**Table 2:** One-sample *t*-test on monthly As (μg/L) concentrations and physico-chemical parameters at the water sampling stations in Langat River during 2005–2014.

Water Stations	Parameters	N	t	df	Sig. (2-Tailed)	Mean Difference	95% Confidence Interval of the Difference	
							Lower	Upper
1L03	As (μg/L)	120	22.19	119	1 × 10^−3^	3.64	3.31	3.96
	DO (mg/L)	120	89.06	119	1 × 10^−13^	5.85	5.72	5.98
	pH	120	232.50	119	1 × 10^−13^	6.98	6.92	7.04
	Temp °C	120	227.87	119	1 × 10^−13^	28.52	28.27	28.77
	SAL (ppt)	120	2.13	119	0.04	0.12	0.01	0.24
	DS (mg/L)	120	19.02	119	1 × 10^−13^	72.82	65.24	80.40
1L15	As (μg/L)	120	18.06	119	1 × 10^−3^	4.74	4.22	5.26
	DO (mg/L)	120	71.15	119	1 × 10^−13^	6.66	6.47	6.84
	pH	120	241.10	119	1 × 10^−13^	7.05	6.99	7.11
	Temp °C	120	253.52	119	1 × 10^−13^	27.15	26.94	27.36
	SAL (ppt)	120	23.70	119	1 × 10^−13^	0.05	0.05	0.06
	DS (mg/L)	120	18.67	119	1 × 10^−13^	57.29	51.22	63.37
1L05	As (μg/L)	120	21.16	119	1 × 10^−3^	4.96	4.49	5.42
	DO (mg/L)	120	57.26	119	1 × 10^−13^	5.54	5.35	5.73
	pH	120	275.22	119	1 × 10^−13^	7.02	6.96	7.07
	Temp °C	120	189.24	119	1 × 10^−13^	28.15	27.85	28.44
	SAL (ppt)	120	29.33	119	1 × 10^−13^	0.07	0.06	0.07
	DS (mg/L)	120	23.50	119	1 × 10^−13^	75.55	69.18	81.91
1L25	As (μg/L)	120	27.70	119	1 × 10^−3^	1.57	1.46	1.69
	DO (mg/L)	120	119.41	119	1 × 10^−13^	6.91	6.79	7.02
	pH	120	185.93	119	1 × 10^−13^	29.46	29.15	29.78
	Temp °C	120	47.90	119	1 × 10^−13^	0.03	0.03	0.03
	SAL (ppt)	120	33.29	119	1 × 10^−13^	37.46	35.23	39.68
	DS (mg/L)	120	119.41	119	1 × 10^−13^	6.91	6.79	7.02

**Table 3:** As (μg/L) concentration at water stations in the Langat River during 2005–2014.

Water Stations	Parameter	N	Minimum	Maximum	Mean	Std. Deviation
1L15	As (μg/L)	120	1.00	21.94	4.74	2.87
	DO (mg/L)	120	3.31	8.67	6.66	1.02
	pH	120	5.67	7.92	7.05	0.32
	Temp °C	120	24.67	30.61	27.15	1.17
	SAL (ppt)	120	0.02	0.13	0.05	0.02
	DS (mg/L)	120	20.00	185.00	57.29	33.62
1L05	As (μg/L)	120	1.00	15.04	4.96	2.57
	DO (mg/L)	120	2.39	7.77	5.54	1.06
	pH	120	5.84	7.73	7.02	0.28
	Temp °C	120	24.67	33.00	28.15	1.63
	SAL (ppt)	120	0.03	0.13	0.07	0.03
	DS (mg/L)	120	28.00	179.00	75.55	35.22
1L03	As (μg/L)	120	0.98	10.60	3.64	1.80
	DO (mg/L)	120	3.55	7.62	5.85	0.72
	pH	120	6.07	8.28	6.98	0.33
	Temp °C	120	25.26	32.34	28.52	1.37
	SAL (ppt)	120	0.02	7.00	0.12	0.63
	DS (mg/L)	120	26.00	265.00	72.82	41.95
1L25	As (μg/L)	120	0.94	3.88	1.57	0.62
	DO (mg/L)	120	4.68	8.84	6.91	0.63
	pH	120	24.17	34.28	29.46	1.74
	Temp °C	120	0.02	0.06	0.03	0.01
	SAL (ppt)	120	18.00	83.00	37.46	12.33
	DS (mg/L)	120	4.68	8.84	6.91	0.63
Average	As (μg/L)	120	0.98	12.87	3.73	1.97
	DO ^4^ (mg/L)	120	3.48	8.23	6.24	0.86
	pH ^4^	120	10.44	14.55	12.63	0.67
	Temp ^5^ °C	120	18.66	24.00	20.96	1.05
	SAL ^6^ (ppt)	120	4.52	22.57	9.43	3.25
	DS ^7^ (mg/L)	120	19.67	159.46	53.14	27.86
MOH ^1^	As (μg/L)		10			
WHO ^2^	As (μg/L)		10			
USEPA ^3^	As (μg/L)		150			

**Note:** ^1^ Drinking water quality standard proposed by Ministry of Health Malaysia [53]; ^2^ Guidelines for drinking water quality proposed by the World Health Organization [54]; ^3^ Toxic reference value proposed by the United States Environmental Protection Agency [55]. ^4, 5, 6, 7^ Mean physico-chemical parameters’ value is within the class I category by Dept. of Environment Malaysia [56].

**Table 4:** Correlation analysis between As (μg/L) and physico-chemical parameters in the Langat River.

Parameters	Correlation	As (μg/L)	DO (mg/L)	pH	Temp °C	SAL (ppt)	DS (mg/L)
As (μg/L)	Pearson Correlation	1					
	Sig. (1-tailed)						
	N	120					
DO (mg/L)	Pearson Correlation	−0.524 **	1				
	Sig. (1-tailed)	4.2 × 10^−10^					
	N	120	120				
pH	Pearson Correlation	0.096	0.121	1			
	Sig. (1-tailed)	0.149	0.095				
	N	120	120	120			
Temp °C	Pearson Correlation	0.229 **	−0.304 **	−0.047	1		
	Sig. (1-tailed)	0.006	3.61 × 10^−4^	0.307			
	N	120	120	120	120		
SAL (ppt)	Pearson Correlation	0.092	−0.052	0.072	−0.002	1	
	Sig. (1-tailed)	0.158	0.285	0.219	0.491		
	N	120	120	120	120	120	
DS (mg/L)	Pearson Correlation	0.704 **	−0.648 **	0.112	0.227 **	0.156 *	1
	Sig. (1-tailed)	1 × 10^−13^	1.01 × 10^−13^	0.112	0.006	0.045	
	N	120	120	120	120	120	120

Note: ** Correlation is significant at the 0.01 level (1-tailed); * Correlation is significant at the 0.05 level (1-tailed).

## Data Availability

The data presented in this study are available upon reasonable request from the corresponding author.

## Supplementary Materials

The following are available online at https://www.mdpi.com/article/10.3390/ijerph18157997/s1: the Six steps of Gradient Boosted Trees (GBT) model’s processes (Figure S1. Overview relative error; Figure S2. Overview runtimes (ms), Table S1. Data used for the Gradient Boosted Trees (GBT) model, Table S2. Comparison of Absolute Error, Root Mean Square, Relative Error, Correlation and Squared Error with Different Models, Table S3. Factors that support and contradict the prediction of each station for the predicted As value for December 2024).
