# Supplementary material for: Predicting Arsenic (As) Exposure on Human Health for Better Management of Drinking Water Sources
Source: Int J Environ Res Public Health. 2021 Jul 28;18(15):7997. doi: 10.3390/ijerph18157997 (PMC8345792; doi:10.3390/ijerph18157997)
Supplement: Supplementary file 1 [file ijerph-18-07997-s001.zip › ijerph-1254548-supplementary.pdf]

Supplemetary Materials

Six steps of the Gradient Boosted Trees (GBT) processes

Step 1: Basic Pre-processing

i. Load and Process of Data: Loads the data set and performing some basic pre-processing tasks like cleaning the data. Delivers all labelled data points as well unlabeled ones for which the model should be applied later on. There are 704 raw data from the year January 2005 until December 2014 that are collected from 8 stations but only 472 data are chosen from only 4 stations because of the data size of 1L07, 1L11, 1L14 and 1L16 are not chosen as each only has 58 data in which the size is too small. The confirmed station order correctly from upstream to downstream is as follows: 1L15(120 data), 1L05 1(120 data), 1L03 (119 data), 1L25 (113 data). Firstly, the redundant values from the same month and year are removed so that in one month of the same year has only one value and the sample is ensured to be collected from the same date from each selected station. Secondly, missing data is inserted with the average value. Missing data are found in only at certain month and year in each station: 1L03 (November and December 2010 and 2011; August 2012), 1L15 (November and December 2010 and 2011), 1L05 1(November and December 2010 and 2011), 1L25 (November and December 2010; March, May, June, August, September, November and December 2014). Missing data occurred mostly in the year 2010 and 2011 and 2014 might be due to the severe flood incidents almost all over Malaysia. Create validation set: Creates a training and a validation set based on 70/30 rules based on 472 selected data. The validation set is used in a robust multiple hold-out performance calculation.

ii. Create validation set: Creates training and a validation set based on 70/30 rules based on 472 selected data. The validation set is used in a robust multiple hold-out performance calculation.

iii. Basic feature engineering: perform some basic feature engineering and pre-processing such as missing values handling or encoding. Text columns is handled later on.

Step 2: Feature engineering and modelling

i. Handling Text Column: Handles text columns if desired and stores the text processing model.

ii. Automatic Feature Engineering: Performs automatic feature engineering if desired. This happens in addition to the basic feature engineering done before (e.g. text processing, data handling, encoding, etc.).

iii. Train Model: Performs the actual model training and automatic hyperparameter tuning (i.e. parameter optimization).

Step 3: Transform validation and scoring data

i. Transform validation data: Transform the validation data (i.e. known target value) using the same preprocessing and features.

ii. Transform scoring data: Transform the scoring data (i.e. no known target value) using the same preprocessing and features.

Step 4: Scoring, validation, explanations, weights and simulator

i. Create predictions and explanations: Applies the model on the validation and the scoring data sets for scoring. Also explains the predictions and calculates model-specific weights. The following is the assumption made:

   a. One year is 365 days, the prediction is for 10 years later, and thus the days' difference is set at a constant of 3650 days multiply by its coefficient weight of 0.228.

b. As L15, L05, L03, and L25 in the correct order are from upstream to downstream, when it is multiplied with the coefficient weight, they are acknowledged as Station 1-4.
c. Out of 472 data obtained from the 4 stations, the GBT model has randomly selected approximately 40-50% of the entire data as a hold-out set (Table S1).

Table S1. Data used for the Gradient Boosted Trees (GBT) model.

Station No.	Total Data Collection	Total Data Selected by GBT on random basis
L15	120	50
L05	120	46
L03	119	49
L25	113	56

ii. Validate model: Performs a multiple hold-out set validation with a robust estimation which provides similar quality of performance estimations than cross-validation with smaller runtimes.
iii. Create model simulator: Creates the model simulator. The weight of each attribute is applied here. The following is the weight by correlation applied to each parameter by Gradient Boosted Trees (GBT).
  a. Month of the year in the sample date: 0.022.
  b. Year in the sample date: 0.227.
  c. Station: 0.521
  d. Days Difference: 0.228

<u>Step 5: Production model</u>

i. Create production model: Creates a final production model by training a model with the same parameters on the combined training and validation data sets. Note that the predicted values are done based on a different set of data's trained.
  a. To predict Jan 2015 As a value, only Jan 2005 data is used as the dataset.
  b. To predict Jan 2016 As a value, data from Jan 2005 until Jan 2006 is used as the dataset.
  c. To predict Jan 2024 As a value, data from Jan 2005 until Jan 2014 is used as the dataset.

<u>Step 6: Process Result</u>

i. Collect runtimes and add annotations to the results. Finally, deliver all result to the result ports. Fig. S1 shows the overview of relative errors and Fig. S2 shows the runtime (ms) of all 6 top models out of 8 that are being modelled in which GBT scores the best.

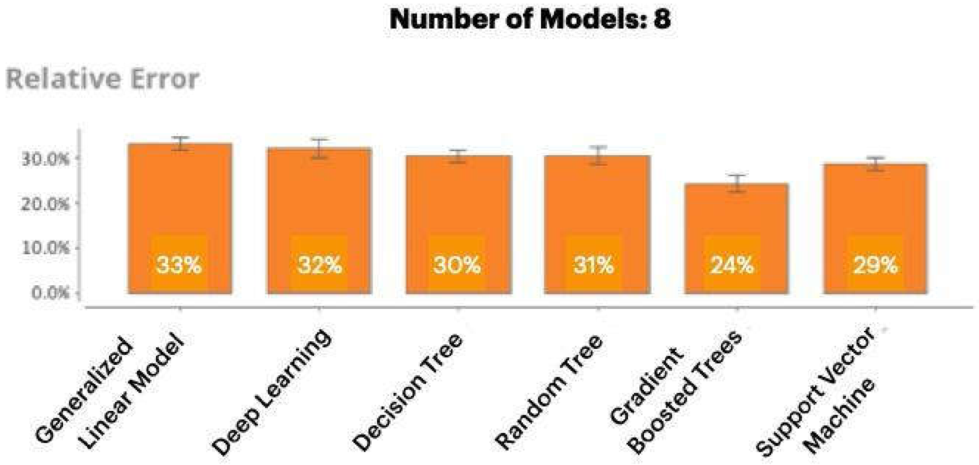

Figure S1. Overview relative error.

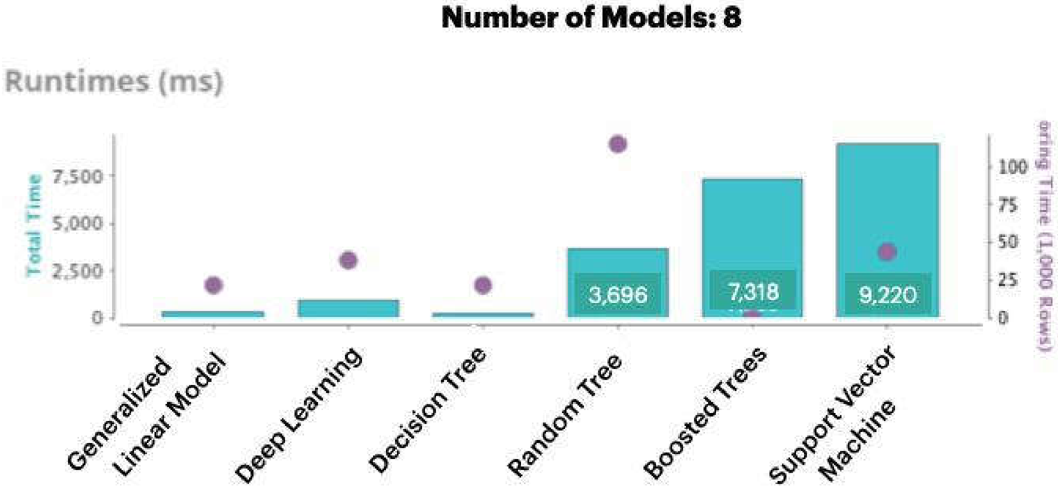

Figure S2. Overview runtimes (ms).

Table S2 shows the comparison of absolute error, root mean square, relative error, correlation and squared error with different models. Out of the top 6 models, GBT has the lowest relative error which is 24.3%, followed by SVM (28.7%). GBT is also having the lowest absolute error (1.199), root mean square (1.869), and squared error (3.766) while GBT has the highest correlation which is 0.763 among the top 6 models selected for modelling.

Table S2. Comparison of Absolute Error, Root Mean Square, Relative Error, Correlation and Squared Error with Different Models.

Model	Absolute Error	Root Mean Square Error	Relative Error	Correlation	Squared Error
Generalized Linear Model	1.528	2.259	33.4%	0.615	5.522
Deep Learning	1.53	2.122	32.3%	0.677	4.927
Decision Tree	1.535	2.38	30.4%	0.547	6.169
Random Forest	1.44	2.156	30.6%	0.705	5.108
Gradient Boosted Trees	1.199	1.869	24.3%	0.763	3.766
Support Vector Machine	1.393	2.238	28.7%	0.651	5.521

There are certain factors that support and contradict the prediction of each station for the predicted As value on Dec 2024 as shown in Table 3. The predicted values of As on that particular month and year in the four respective stations are 1L15: 3.587, 1L05: 3.947, 1L03: 3.667, and 1L25: 1.258. Stations 1L03, 1L05 and 1L15 are the factors that support prediction but not 1L25. This is due to Stations 1L03, 1L05 and 1L15 support the increment while station 1L25 support the decrement of the As value as compared to the average value of the previous data. Another factor that supports the prediction is the sample date in which the samples are collected on the first day of the week in all four stations. Other remaining factors that contradicted the predictions are including the prediction date, the month of the year, the first and third day of the week, the first month of the quarter and the third quarter of the year.

Table S3. Factors that support and contradict the prediction of each station for the predicted As value on Dec 2024.

**Stations**	**1L03**	**1L15**	**1L05**	**1L25**
Prediction	3.667	3.587	3.947	1.258
Important Factors: STA No	0.201	0.250	0.390	-0.930
Important Factors: days_diff (SMP-DAT, Today)	-0.202	-0.203	-0.201	-0.019
Important Factors: SMP-DAT: month_of_year	-0.181	-0.186	0.083	-0.018
Important Factors: SMP-DAT: day_of_week = 1	0.080	0.085	-0.083	0.008
Important Factors: SMP-DAT: day_of_week = 3	-0.065	-0.057	-0.063	-0.006
Important Factors: SMP-DAT: month_of_quarter = 1	-0.064	-0.053	-0.060	-0.006

Note: (+) Positive number refers supporting factor; (-) Negative number refers contradicting factor.
